# Effect of Sputtering Oxygen Partial Pressure on the Praseodymium-Doped InZnO Thin Film Transistor Using Microwave Photoconductivity Decay Method

**DOI:** 10.3390/mi12091044

**Published:** 2021-08-29

**Authors:** Huansong Tang, Kuankuan Lu, Zhuohui Xu, Honglong Ning, Dengming Yao, Xiao Fu, Huiyun Yang, Dongxiang Luo, Rihui Yao, Junbiao Peng

**Affiliations:** 1 State Key Laboratory of Luminescent Materials and Devices, Institute of Polymer Optoelectronic Materials and Devices, South China University of Technology, Guangzhou 510640, China; 201865320283@mail.scut.edu.cn (H.T.); kk-lu@foxmail.com (K.L.); 201866320374@mail.scut.edu.cn (D.Y.); 201630343721@mail.scut.edu.cn (X.F.); 201830320362@mail.scut.edu.cn (H.Y.); psjbpeng@scut.edu.cn (J.P.); 2 Guangxi Key Lab of Agricultural Resources Chemistry and Biotechnology, Yulin Normal University, Yulin 537000, China; xzh21@ylu.edu.cn; 3 Institute of Semiconductors, South China Normal University, Guangzhou 510631, China; luodx@gdut.edu.cn; 4 School of Materials and Energy, Guangdong University of Technology, Guangzhou 510006, China

**Keywords:** praseodymium-doped InZnO, oxygen partial pressure, microwave photoconductivity decay, thin film transistor

## Abstract

The praseodymium-doped indium-zinc-oxide (PrIZO) thin film transistor (TFT) shows broad application prospects in the new generation of display technologies due to its high performance and high stability. However, traditional device performance evaluation methods need to be carried out after the end of the entire preparation process, which leads to the high-performance device preparation process that takes a lot of time and costs. Therefore, there is a lack of effective methods to optimize the device preparation process. In this paper, the effect of sputtering oxygen partial pressure on the properties of PrIZO thin film was studied, and the quality of PrIZO thin film was quickly evaluated by the microwave photoconductivity decay (µ-PCD) method. The μ-PCD results show that as the oxygen partial pressure increases, the peak first increases and then decreases, while the D value shows the opposite trend. The quality of PrIZO thin film prepared under 10% oxygen partial pressure is optimal due to its low localized defect states. The electric performance of PrIZO TFTs prepared under different oxygen partial pressures is consistent with the μ-PCD results. The optimal PrIZO TFT prepared under 10% oxygen partial pressure exhibits good electric performance with a threshold voltage (*V_th_*) of 1.9 V, a mobility (*µ_sat_*) of 24.4 cm^2^·V^−1^·s^−1^, an *I_on_*/*I_of_*_f_ ratio of 2.03 × 10^7^, and a subthreshold swing (*SS*) of 0.14 V·dec^−1^.

## 1. Introduction

With the development of semiconductor devices, thin film transistors (TFTs) are widely used in many display fields [1,2,3,4,5]. As the active layer of TFTs, metal oxide semiconductor materials have the advantages of high mobility and high optical transparency, which shows great application potential [6,7,8,9,10]. In fact, metal oxide semiconductor materials represented by indium-gallium-zinc oxide (IGZO) have been partially applied [11,12,13]. However, IGZO technology has the problem of being sensitive to external environmental factors such as light, water and oxygen [14,15]. In order to address these problems, the research of adding various dopants to indium zinc oxide (IZO) to improve stability has attracted people’s attention [16,17,18,19]. The praseodymium-doped IZO (PrIZO) TFTs have a significant inhibitory effect on light-induced instability and improve the stability of bias illumination [20,21]. In the meantime, the praseodymium-doped IZO TFTs improve the thermal stability of the devices [22,23,24,25]. In addition, praseodymium doping also improves the aging effect of the device channel exposed to the external environment [26].

Radio frequency (RF) magnetron sputtering is an efficient thin film deposition technology, which has been widely used in industrial production and scientific research [27,28]. During the deposition process, due to the small working window of the RF magnetron sputtering equipment, the condition of sputtering oxygen partial pressure greatly affects the quality of the semiconductor thin film [29,30,31]. Therefore, the optimization of sputtering oxygen partial pressure is very essential. The quality of semiconductor thin films can be evaluated by the transfer curves of TFTs prepared under different conditions, but the device preparation process obviously requires a lot of time and costs. As a non-destructive and non-contact method, the microwave photoconductive decay (µ-PCD) method can quickly evaluate the quality of semiconductor thin films by obtaining defect status information of semiconductor thin films. Therefore, the μ-PCD method can be used as an effective idea for optimizing the device preparation process, which greatly reduces the time and cost of the entire device preparation process [32,33,34,35]. In addition, positron annihilation spectroscopy (PAS) can also be used to characterize defects. It mainly uses the positron probe to annihilate the electrons in the material, and by detecting the information of the γ photons generated by the annihilation, so as to achieve the purpose of studying the microscopic defects of the material [36,37].

In this paper, the effect of sputtering oxygen partial pressure on PrIZO thin film properties was studied, and PrIZO TFTs were prepared under different oxygen partial pressures. The quality of PrIZO thin film was quickly evaluated by the μ-PCD method and the results found that the thin film has a low defect state under 10% oxygen partial pressure. PrIZO TFT prepared under 10% oxygen partial pressure exhibits good electric performance with a threshold voltage (*V_th_*) of 1.9 V, a mobility (*µ_sat_*) of 24.4 cm^2^·V^−1^·s^−1^, an *I_on_*/*I_off_* ratio of 2.03 × 10^7^, and a subthreshold swing (*SS*) of 0.14 V·dec^−1^. Compared with TFTs prepared under other oxygen partial pressures, the performance of TFT prepared under 10% oxygen partial pressure is the optimal, which well confirms the μ-PCD results. Therefore, the μ-PCD method can effectively evaluate the quality of the semiconductor film and provides an idea for optimizing the preparation of the devices.

## 2. Materials and Methods

PrIZO thin film was deposited by RF magnetron sputtering using a target (Pr:In:Zn = 0.1:1:1 wt %). The carrier gas of RF magnetron sputtering is Ar. A 50-nm-thick PrIZO thin film was deposited on an alkali-free glass (1.0 × 1.0 cm^2^) under various oxygen partial pressures (0%, 5%, 10% and 20%). The sputtering power and sputtering pressure were maintained at 80 W and 0.7 Pa, respectively. In addition, PrIZO thin film was not annealed after deposition. The purpose is to eliminate the effect of annealing temperature and more completely study the effect of sputtering oxygen partial pressure on thin film properties.

**Figure 1:**
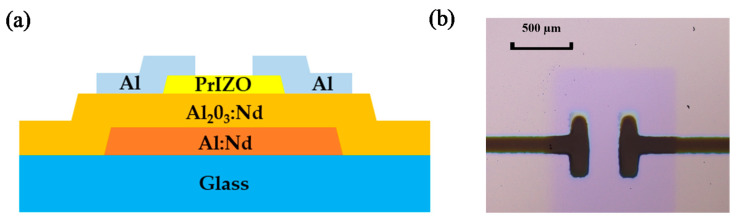
Praseodymium-doped indium-zinc-oxide (PrIZO) thin film transistor (TFT) (**a**) schematic cross-sectional view, (**b**) micrograph.

The cross-sectional schematic view and the micrograph of PrIZO TFT are shown in Figure 1a, b, respectively. PrIZO TFT was manufactured on an alkali-free glass substrate (1.0 × 1.0 cm^2^). The gate electrode is 100-nm-thick Al-Nd alloy covered with 200-nm-thick anodized AlO_x_: Nd insulator [38]. A 25-nm-thick PrIZO thin film channel layer was continuously deposited through a mask by RF magnetron sputtering. During the deposition process, the working environment of the equipment was room temperature, and the sputtering power and sputtering pressure were set to 80 W and 0.7 Pa, respectively. After the deposition, the device was annealed at 150 °C in air for one hour. Finally, using the DC magnetron sputtering mode, a 200-nm-thick aluminum film was sputtered through a mask (channel width/length = 500 µm/400 µm) to form source/drain electrodes. The sputtering power and sputtering pressure were maintained at 80 W and 1.3 Pa, respectively.

The micrograph of PrIZO TFT was measured by the optical microscope (MSHOT, MJ30, Guangzhou, Guangdong, China). The surface morphology of PrIZO thin film was characterized by atomic force microscopy (AFM) (BY3000, Being Nano-Instruments, Guangzhou, China). The phase of PrIZO thin film was characterized by X-ray diffraction (XRD) (Empyrean Nano edition, PANalytical, Almelo, The Netherlands). The optical properties of PrIZO thin film were studied by a UV-VIS spectrophotometer (Shimadzu UV-3600, Kyoto, Japan). The chemical changes of PrIZO thin films were detected by X-ray photoelectron spectroscopy (XPS) measurement (ESCALAB250Xi, Thermo Fisher Scientific, Waltham, MA, USA). The quality of PrIZO thin film was evaluated by the µ-PCD measurement system (LTA-1620SP, Kobelco, Japan). The electric performance of PrIZO TFT was measured using semiconductor analyzer (Agilent 4155C) in dark and air environments.

## 3. Results and Discussion

### 3.1. AFM Analysis

**Figure 2:**
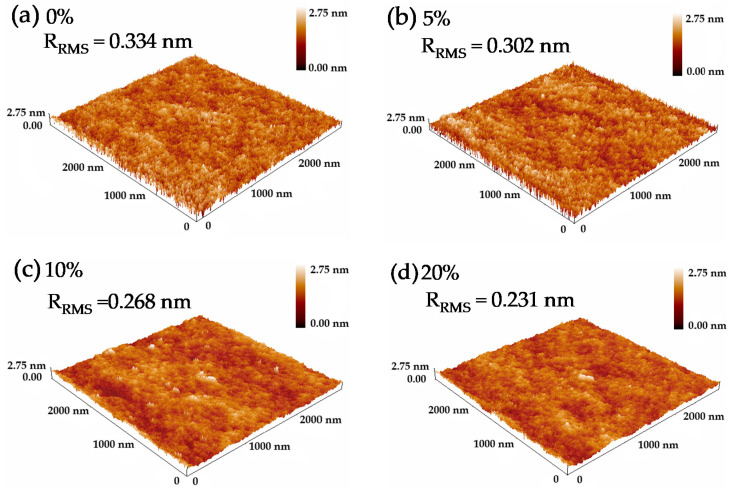
Atomic force microscopy (AFM) images of PrIZO thin films with different oxygen partial pressures: (**a**) 0%, (**b**) 5%, (**c**) 10%, (**d**) 20%.

Figure 2 shows AFM images (3.0 × 3.0 µm^2^) of PrIZO thin films with different oxygen partial pressures. From Figure 2, the root-mean-square roughness (R_RMS_) of PrIZO thin films is as low as 0.334 nm, which indicates all films are smooth. As the oxygen partial pressure increases, the R_RMS_ of PrIZO thin film gradually decreases. The R_RMS_ of PrIZO thin film prepared under 20% oxygen partial pressure is reduced to 0.231 nm. Therefore, the increase in oxygen partial pressure improves the uniformity of PrIZO thin film.

### 3.2. XRD Analysis

**Figure 3:**
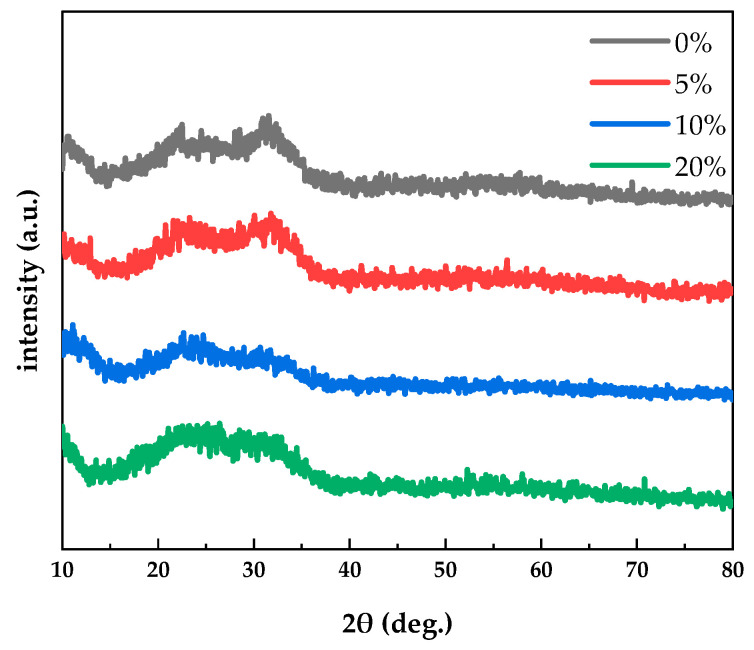
X-ray diffraction (XRD) patterns of PrIZO thin films prepared under various oxygen partial pressures.

Figure 3 shows XRD patterns of PrIZO thin films prepared under various oxygen partial pressures. From Figure 3, all PrIZO thin films are amorphous under various oxygen partial pressures. Besides, a broad peak between 20° and 35° could be found due to the amorphous nature of the glass substrate. There are large differences in the lattice structure among hexagonal Pr_2_O_3_, cubic In_2_O_3_ and hexagonal wurtzite ZnO, which may lead to the amorphous structure of PrIZO.

### 3.3. Optical Characterization

**Figure 4:**
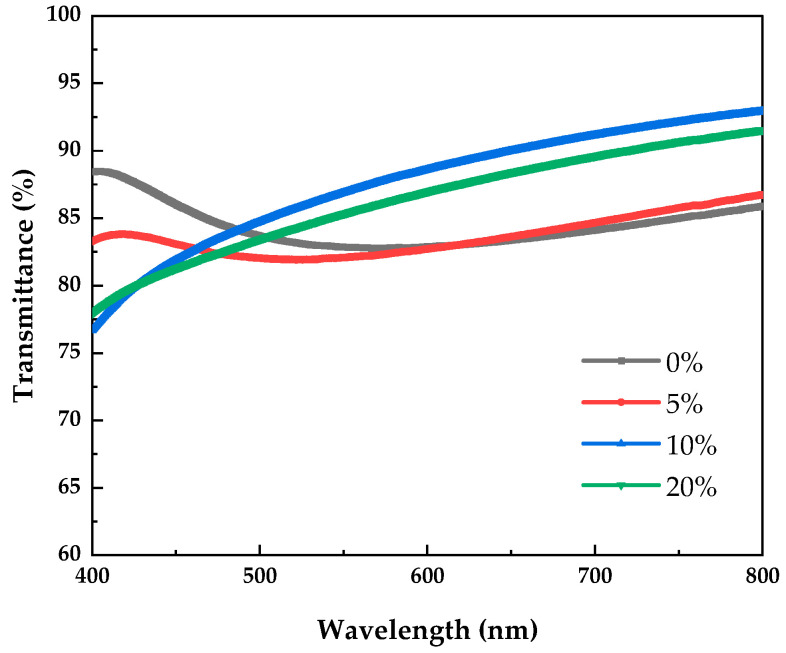
Transmission spectra of PrIZO thin films prepared under various oxygen partial pressures.

Figure 4 shows the transmission spectra of PrIZO thin films prepared under various oxygen partial pressures. The average transmittance of PrIZO thin film in the visible light region is shown in Table 1, the average transmittance of all samples exceeds 83%. In addition, the increase in oxygen partial pressure increases the average transmittance of PrIZO thin film in the visible light region. Especially, the average transmittance of PrIZO thin film reaches 87.6% under 10% oxygen partial pressure. These results show that PrIZO thin films are promising candidates for transparent electronic devices.

The optical band gap $\left(E_{g}\right)$ can be calculated according to Equation (1):

(1) $${\left(\alpha hv\right)}^{2}=A\left(hv-E_{g}\right)$$

where  α is the absorption coefficient, hv is the photon energy and A is a constant. $E_{g}$ is the intercept that extends the linear part of the ${\left(\alpha hv\right)}^{2}$ − hv curve to the abscissa. 

**Figure 5:**
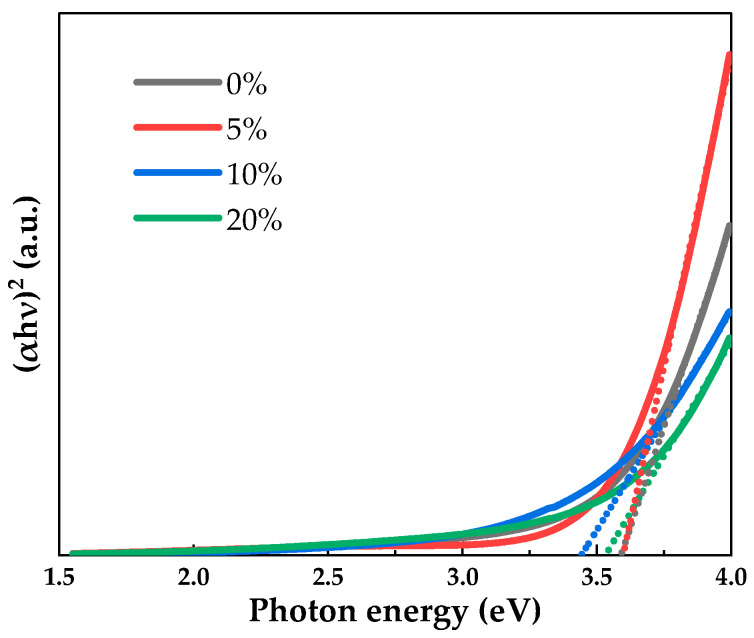
Optical band gap extraction images of PrIZO thin films with different oxygen partial pressures.

Figure 5 shows the optical band gap extraction images of PrIZO thin films with different oxygen partial pressures. The optical band gap is shown in Table 1. The optical band gap of PrIZO thin film at 0% oxygen partial pressure is 3.60 eV. When the oxygen partial pressure increases to 10%, the optical band gap of PrIZO thin film decreases to 3.45 eV. According to the Burstein–Moss model, the change of the optical band gap is related to the carrier concentration, and excess carriers will cause the optical band gap to blue shift [39,40]. The carriers in oxide semiconductors mainly originate from oxygen vacancies. Therefore, according to the following XPS results, it can be well explained that appropriately increasing the oxygen partial pressure can reduce the oxygen vacancies, and the decrease in the carrier concentration provided by the oxygen vacancies leads to a decrease in the optical band gap.

### 3.4. XPS Analysis

**Figure 6:**
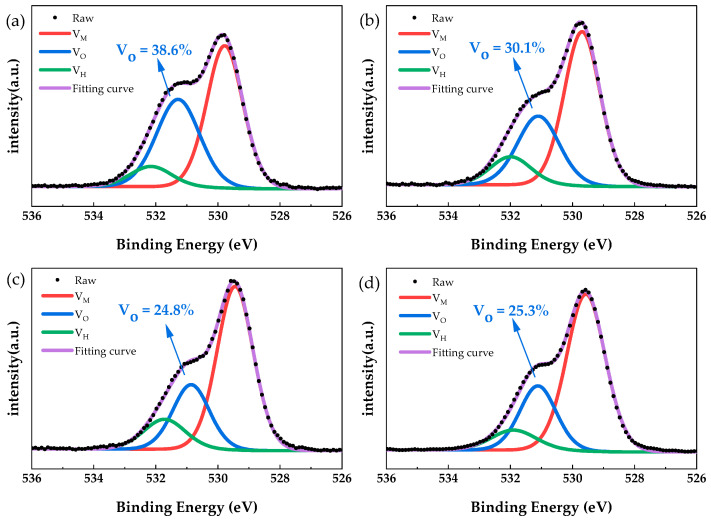
The O 1s spectra of PrIZO thin films with different oxygen partial pressures: (**a**) 0%, (**b**) 5%, (**c**) 10%, (**d**) 20%.

Figure 6 shows the O 1s spectra of PrIZO thin films with different oxygen partial pressures. The O 1s spectra of all films could be well separated into three Gauss–Lorentz components centered at 529.6 ± 0.2 eV (V_M_), 531.1 ± 0.3 eV (V_O_) and 531.9 ± 0.2 eV (V_H_). The three peaks are generally attributed to metal-ions-bonded O^2−^, O^2−^ in the oxygen-deficient region and chemisorbed oxygen, respectively [41,42]. The relative area of the V_O_ peak of PrIZO thin film under 0%, 5%, 10% and 20% oxygen partial pressure is 38.6%, 30.1%, 24.8% and 25.3%, respectively. The decrease in the relative area of the V_O_ peak is related to the decrease of oxygen vacancies, so the increase in oxygen partial pressure can effectively reduce the oxygen vacancies. In addition, compared with PrIZO thin film under 10% oxygen partial pressure, the relative area of the V_O_ peak of the thin film under 20% oxygen partial pressure is slightly increased. Therefore, it is not correct that the higher the oxygen partial pressure, the fewer oxygen vacancies.

### 3.5. Thin Film Performance

The µ-PCD measurement system uses laser irradiation to change the conductivity of semiconductor thin film, which changes the microwave reflectivity. The μ-PCD method can obtain the µ-PCD curve through the change process of microwave reflectivity in the process of photocarrier capture and recombination, and then evaluate the quality of semiconductor thin film by extracting the Peak and D value from the µ-PCD curve.

**Figure 7:**
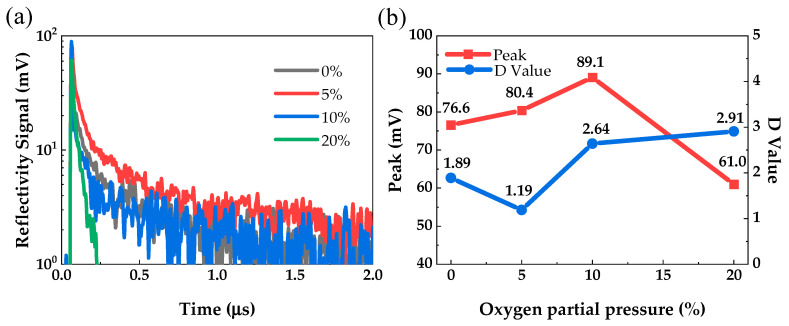
The microwave photoconductivity decay (µ-PCD) results of PrIZO thin films: (**a**) the µ-PCD curves, (**b**) peak and D value.

Figure 7 shows the µ-PCD results of PrIZO thin films prepared under various oxygen partial pressures. From Figure 7a, the µ-PCD curve is composed of three components: peak, fast decay and slow decay. The peak is the maximum microwave reflection signal generated by the increase in the photo-generated carrier density during the laser pulse radiation process, which is related to the density of the conduction band tail state [43]. The rapid decay is related to the recombination process of photogenerated carriers in the deep localized states, the peak can also be used to evaluate deep traps due to the short decay time of fast decay. D value is a parameter extracted from the slow decay component which is related to the capture and release of photogenerated carriers in shallow localized states [44]. The higher the peak and the D value, the better the quality of film. 

From Figure 7b, the peak of PrIZO thin film shows a trend of first increasing and then decreasing with the increase of oxygen partial pressure. The result means an appropriate amount of oxygen partial pressure can effectively reduce the deep-level defect density of PrIZO thin film. Compared with other oxygen partial pressures, the peak of PrIZO thin film under 10% oxygen partial pressure is the maximum. In addition, the D value of PrIZO thin film shows the opposite trend with the increase of oxygen partial pressure. The D value of PrIZO thin film under 20% oxygen partial pressure is the maximum. PrIZO thin film has a low shallow-level defect density under 20% oxygen partial pressure, but its deep-level defect density is greatly increased. Therefore, considering the peak and D value comprehensively, it can be considered that the quality of PrIZO thin film is optimal under 10% oxygen partial pressure.

**Figure 8:**
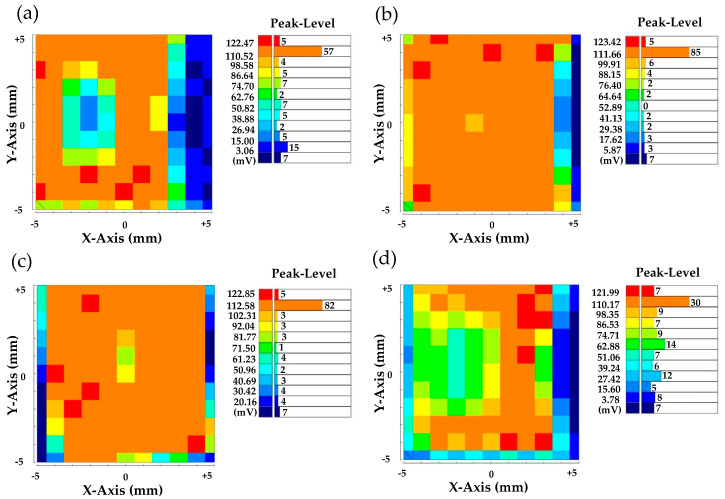
Peak mapping results of (**a**) the unannealed PrIZO thin film and PrIZO thin films annealed at (**b**) 150 °C, (**c**) 250 °C, (**d**) 450 °C.

In order to further optimize the annealing conditions of PrIZO TFTs, the peak mapping results of PrIZO thin films annealed at different temperatures in air for one hour were studied using the µ-PCD method, as shown in Figure 8. The larger the number of color units with large peaks, the fewer defects of PrIZO thin film. Compared with the unannealed film, the quality of PrIZO thin film annealed at 150 °C or 250 °C is improved, but the quality of the film annealed at 450 °C is inferior. This may be an excessively high annealing temperature leading to increased film defects. Although the peak mapping results of PrIZO thin film at 150 °C and 250 °C are similar, since a lower annealing temperature can better study the effect of oxygen partial pressure, 150 °C is chosen as the annealing temperature of PrIZO TFTs.

### 3.6. Device Electric Performance

**Figure 9:**
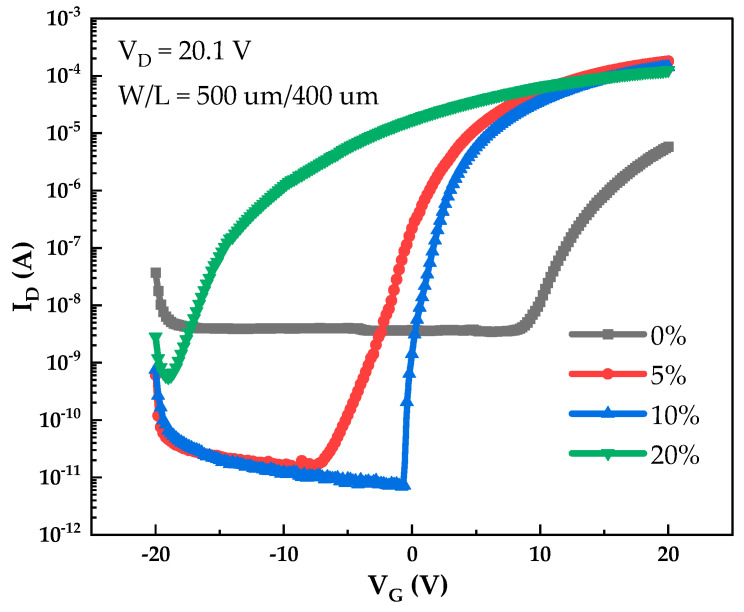
Transfer curves of PrIZO TFTs with different oxygen partial pressures.

Figure 9 shows the transfer curves of PrIZO TFTs with different oxygen partial pressures. The electric parameters of PrIZO TFTs are extracted from the transfer curves, as shown in Table 2. The optimal PrIZO TFT under 10% sputtering oxygen partial pressure exhibits good electric performance with a *V_th_* of 1.9 V, a *µ_sat_* of 24.4 cm^2^·V^−1^·s^−1^, an *I_on_*/*I_off_* ratio of 2.03 × 10^7^, and an *SS* of 0.14 V·dec^−1^. It is found that the peak signal of the μ-PCD method has a strong correlation with the field-effect mobility of TFTs [33]. The μ-PCD results show that the peak signal of the film is the largest under 10% oxygen partial pressure, and the field-effect mobility of the corresponding TFT device is also the largest. This may be because there are fewer defects in the thin film under 10% oxygen partial pressure, which reduces carrier scattering and improves the field-effect mobility of the device.

## 4. Conclusions

In summary, the effect of sputtering oxygen partial pressure on the properties of PrIZO thin film was studied, and the quality of PrIZO thin film was quickly evaluated by the µ-PCD method. The µ-PCD analysis shows that the quality of PrIZO thin film prepared under 10% oxygen partial pressure is optimal. Under 10% sputtering oxygen partial pressure, the average transmittance of PrIZO thin film reaches 87.6% in the visible light region, and the optimal PrIZO TFT exhibits good electric performance with a *V_th_* of 1.9 V, a *µ_sat_* of 24.4 cm^2^·V^−1^·s^−1^, an *I_on_*/*I_off_* ratio of 2.03 × 10^7^, and an *SS* of 0.14 V·dec^−1^. The µ-PCD method can be used to quickly evaluate the quality of semiconductor thin film, which greatly reduces the time and cost of the device preparation process. Therefore, this article provides an idea for optimizing the device preparation process. 

## Figures and Tables

**Table 1:** Optical properties of praseodymium-doped indium-zinc-oxide (PrIZO) thin films with different oxygen partial pressures.

Oxygen Partial Pressure (%)	Average Transmittance (%)	Optical Band Gap (eV)
0	84.7	3.60
5	83.6	3.60
10	87.6	3.45
20	86.4	3.53

**Table 2:** Electric parameters of PrIZO TFTs.

Oxygen Partial Pressure (%)	*V_th_* (V)	*µ_sat_* (cm^2^·V^−1^·s^−1^)	*I_on_/I_off_*	*SS* (V·dec^−1^)
0	12.6	9.3	1.69 × 10^3^	0.63
5	0.3	22.7	1.31 × 10^7^	0.28
10 20	1.9 −10.7	24.4 16.5	2.03 × 10^7^ 2.25 × 10^5^	0.14 0.53

## Data Availability

Not applicable.
